# The NO-cGMP-PKG Signaling Pathway Coordinately Regulates ERK and ERK-Driven Gene Expression at Pre- and Postsynaptic Sites Following LTP-Inducing Stimulation of Thalamo-Amygdala Synapses

**DOI:** 10.1155/2010/540940

**Published:** 2011-02-20

**Authors:** Junli Ping, Glenn E. Schafe

**Affiliations:** ^1^Department of Psychology, Yale University, 2 Hillhouse Avenue, Box 208205, New Haven, CT 06520, USA; ^2^Interdepartmental Neuroscience Program, Yale University, 2 Hillhouse Avenue, Box 208205, New Haven, CT 06520, USA

## Abstract

Long-term potentiation (LTP) at thalamic input synapses to the lateral nucleus of the amygdala (LA) has been proposed as a cellular mechanism of the formation of auditory fear memories. We have previously shown that signaling via ERK/MAPK in both the LA and the medial division of the medial geniculate nucleus/posterior intralaminar nucleus (MGm/PIN) is critical for LTP at thalamo-LA synapses. Here, we show that LTP-inducing stimulation of thalamo-LA inputs regulates the activation of ERK and the expression of ERK-driven immediate early genes (IEGs) in both the LA and MGm/PIN. Further, we show that pharmacological blockade of NMDAR-driven synaptic plasticity, NOS activation, or PKG signaling in the LA significantly impairs high-frequency stimulation-(HFS-) induced ERK activation and IEG expression in both regions, while blockade of extracellular NO signaling in the LA impairs HFS-induced ERK activation and IEG expression exclusively in the MGm/PIN. These findings suggest that NMDAR-driven synaptic plasticity and NO-cGMP-PKG signaling within the LA coordinately regulate ERK-driven gene expression in both the LA and the MGm/PIN following LTP induction at thalamo-LA synapses, and that synaptic plasticity in the LA promotes ERK-driven transcription in MGm/PIN neurons via NO-driven “retrograde signaling”.

## 1. Introduction

Fear conditioning is a type of associative learning that is widely studied as a model of learning and memory across a variety of species. Fear conditioning has been extensively characterized at the behavioral level, particularly auditory fear conditioning, in which a tone (CS; conditioned stimulus) is paired with footshock (US; unconditioned stimulus). In brief, auditory fear conditioning is thought to involve transmission and integration of sensory information from CS and US pathways within the lateral nucleus of the amygdala (LA), where alterations in synaptic transmission are believed to encode key aspects of the learning [1–3]. In support of this hypothesis, auditory fear conditioning has been shown to regulate neural activity in the LA; that is, LA neurons respond weakly to a tone CS before conditioning, but respond in a robust manner to the CS after fear conditioning [4, 5]. 

Long-term potentiation (LTP), an experimental model of synaptic plasticity, is widely believed to be a potential mechanism by which fear conditioning promotes synaptic alterations in the LA [1, 6]. In support of this hypothesis, LTP has been demonstrated in each of the major sensory input pathways that are known to be important for auditory fear conditioning [7–10]. Further, LTP induction at auditory thalamic inputs to the LA has been shown to augment the processing of natural auditory information in the LA [11], and auditory fear conditioning induces neurophysiological changes in the LA that are similar to artificial LTP induction [5, 12]. Finally, auditory fear conditioning and LTP have been shown to be subserved by similar stimulus contingencies [13] and pharmacological mechanisms [14, 15]. 

While the relationship between LTP in the LA and fear conditioning has been extensively studied, we have only begun to explore the underlying molecular mechanisms by which LTP promotes synaptic changes in the LA. Recent studies employing *in vitro* slice recording methods have pointed to a role for a number of intracellular signaling pathways in LTP at thalamic input synapses to the LA, including CaMKII [16], PKA [9, 14], ERK/MAPK [14, 15], and the NO-cGMP-PKG signaling pathway [17, 18]. Very little, however, is known about how these signaling pathways are related to each other or the identity of the downstream nuclear targets of these pathways that promote long-lasting LTP in the LA. Further, the involvement of the NO-cGMP-PKG signaling pathway suggests that LTP at thalamo-amygdala synapses may be characterized by pre- as well as postsynaptic alterations in gene expression and structural plasticity [19–21]. In support of this hypothesis, recent studies from our lab have shown that ERK/MAPK activation in both the LA [22] and in regions of the auditory thalamus that are presynaptic to the LA, including the medial geniculate nucleus and the posterior intralaminar nucleus (MGm/PIN) [23], are critical for long-lasting LTP at thalamo-LA synapses. This pattern of findings collectively suggests that LTP at thalamic input synapses to the LA regulates ERK activation and ERK-driven transcription at both sides of the thalamo-LA synapse.

In the present study, we first used a combination of Western blotting and immunocytochemistry to examine whether LTP-inducing stimulation of thalamo-LA synapses regulates ERK/MAPK activation and ERK-driven gene expression in both the LA and the MGm/PIN. Next, we used pharmacological methods combined with Western blotting to examine the extent to which NMDAR-driven synaptic plasticity and NO-cGMP-PKG signaling at the level of the LA regulates ERK activation and ERK-driven immediate early gene (IEG) expression in both LA and MGm/PIN following LTP-inducing stimulation of thalamo-LA inputs. 

## 2. Materials and Methods

### 2.1. Subjects

Adult male Sprague-Dawley rats (Harlan), weighing 300–325 g, were housed individually in plastic cages and maintained on a 12 : 12 h light/dark cycle. Food and water were provided *ad libitum* throughout the experiment. 

### 2.2. Surgical Procedures

Rats were anesthetized with 40% Urethane (*i.p*. injections at 10 min intervals; total of 1.6 mg/kg) and placed in a stereotaxic frame. The skull was exposed, small holes were drilled over the left LA and/or the MGm/PIN., and the dura was retracted. For stimulation experiments, rats were implanted with a bipolar stimulating electrode into the MGm/PIN. For pharmacology/stimulation experiments, rats were implanted unilaterally with a 23-gauge stainless-steel guide cannula aimed at the LA, and a bipolar stimulating electrode into the ipsilateral MGm/PIN. The coordinates for the LA were: −3.2 mm anterior-posterior, 5.0 mm medial-lateral, −8.0 mm dorsal-ventral relative to Bregma. The coordinates for the MGm/PIN were: −5.6 mm medial-lateral, 2.9 mm medial-lateral, and −6.6 mm dorsal-ventral [24]. All procedures were conducted in accordance with the National Institutes of Health “Guide for the Care and Use of Experimental Animals” and were approved by the Yale University Animal Care and Use Committee.

### 2.3. Electrical Stimulation Experiments

One-half hour after implantation of the stimulation electrode, rats were given LTP-inducing (high-frequency) stimulation (HFS) consisting of three series of theta-patterned 100 Hz tetanic stimulation given once a minute at an intensity of 300 *μ*A, 100 *μ*s, a protocol that reliably induces LTP in the LA [22, 25, 26]. Low-frequency stimulation controls received the same total number of pulses as rats in the HFS group (300 total pulses over 2 min) but at lower frequency (2.5 Hz), a protocol that does not induce LTP in the LA [22]. In all stimulation experiments, current was applied such that it moved from the tip to the tube of the bipolar stimulation electrode. 

### 2.4. Drugs

The selective NR2B antagonist ifenprodil (Sigma, Cat. No. I2892) was dissolved in 2% 2-hydroxypropyl-*β*-cyclodextrin-(HBC-) saline solution in a stock concentration of 2 *μ*g/*μ*L. The selective NOS inhibitor 7-nitroindazole (7-Ni; Calbiochem, Cat. No. 483400) and the NO scavenger 2-(4-carboxyphenyl)-4,4,5,5-tetramethylimidazoline-1-oxyl-3-oxide (c-PTIO; Tocris, Cat. No. 0772) were dissolved in 100% DMSO to a final stock concentration of 4 *μ*g/*μ*L. Prior to infusion into the brain, the drug was diluted 1 : 1 in ACSF. The PKG inhibitor guanosine 3′,5′-cyclic monophosphorothioate, *β*-Phenyl-1,N^2^-etheno-8-bromo-, Rp-Isomer, sodium salt (Rp-8-Br-PET-cGMPS; Calbiochem, Cat. No. 370679) was dissolved in distilled water in a stock concentration of 2 *μ*g/*μ*L. 

### 2.5. Drug Infusions

Cannulated rats were given an intra-LA infusion of either ifenprodil (1.0 *μ*g), 7-Ni (1.0 *μ*g), c-PTIO (1.0 *μ*g), Rp-8-Br-PET-cGMPS (1.0 *μ*g) or the appropriate vehicle solution 30 min following implantation. Each of the drug or vehicle solutions was infused at a volume of 0.5 *μ*L. The cannulas were connected to 1.0 *μ*L Hamilton syringes via polyurethane tubing. The tubing was back-filled with sesame oil, with a small air bubble separating the oil from the drug solution, which was infused bilaterally with an infusion pump at a constant rate of 0.25 *μ*L/min. After infusion, the injector remained in the guide cannula for 1 minute to allow diffusion of the drug from the tip. 

### 2.6. Western Blotting Experiments

Rats were given LTP-inducing HFS 30 min after infusion and sacrificed at the appropriate time point after stimulation by decapitation. For Western blotting experiments, punches containing the LA and MGm/PIN both ipsilateral and contralateral to the side of stimulation were obtained with a 1 mm punch tool (Fine Science Tools) from 400-*μ*m-thick sections taken on a sliding freezing microtome. Punches were manually dounced in 100 *μ*L of ice-cold hypotonic lysis buffer (10 mM Tris-HCl, pH 7.5, 1 mM EDTA, 2.5 mM sodium pyrophosphate, 1 mM phenylmethylsulfonyl fluoride, 1 mM *β*-glycerophosphate, 1% Igepal CA-630, 1% protease inhibitor cocktail (Sigma), and 1 mM sodium orthovanadate). 

Sample buffer was immediately added to the homogenates, and the samples were boiled for 4 min. Homogenates (20 *μ*g/lane) were electrophoresed on 10% Tris-HCl gels and blotted to Immobilon-P (Millipore). 

 To examine phosphorylated (activated) ERK or total ERK, Western blots were blocked in 5% milk and then incubated with an antiphospho-MAPK (1 : 1000; Cell Signaling) or an anti-total MAPK antibody (1 : 1000; Cell Signaling). Blots were then incubated with an antirabbit secondary antibody conjugated to horseradish peroxidase (1 : 20,000; Cell Signaling) and developed using enhanced chemiluminescence (Pierce). Optical densities of the bands were analyzed using NIH ImageJ software. To assess for changes in the activation of ERK/MAPK, phosphorylated kinase levels were normalized to total ERK levels. To confirm that total ERK levels remained constant across infusions, blots were blocked in 5% BSA in TTBS and reincubated in GAPDH antibody (1 : 5000; Abcam). Following incubation with an antimouse secondary antibody conjugated to horseradish peroxidase (1: 20,000; Cell Signaling), blots were developed identically to those processed for phospho-ERK and total ERK. Total ERK levels were then normalized to GAPDH levels for analysis.

 To examine Arc/Arg3.1, Western blots were blocked in TTBS buffer (20 mM Tris-HCl, pH 7.5, 150 mM NaCl, and 0.05 Tween 20) with 5% dry milk and then incubated with Arc antibody (1 : 1000; Santa Cruz Biotechnology). Blots were then incubated with anti-mouse conjugated to horseradish peroxidase (1: 20,000; Cell Signaling). For EGR-1, blots were blocked in TTBS with 5% milk and then incubated with EGR-1 antibody (1 : 1000; Santa Cruz Biotechnology). Blots were then incubated with anti-rabbit antibody conjugated to horseradish peroxidase (1: 20,000; Cell Signaling). For c-Fos, blots were blocked in 4% milk in TTBS and then incubated with c-Fos antibody (1 : 1000; Cell Signaling). Blots were then incubated with anti-rabbit conjugated to horseradish peroxidase (1: 20,000; Cell Signaling). All blots were developed using West Dura chemiluminescence (Pierce laboratories). To control for inconsistencies in loading, optical densities of Arc/Arg3.1, EGR-1 and c-Fos were all normalized to GAPDH protein levels.

### 2.7. Quantification of Western Blots

Densitometry was conducted using NIH ImageJ software. For analysis, data from the ipsilateral side were expressed as a percentage of that from the contralateral side for each rat. Data were analyzed using paired-sample *t*-test (ipsilateral versus contralateral). 

### 2.8. Immunohistochemistry

For immunohistochemical experiments, rats were given either HFS or LFS. Two hours following stimulation, rats were rapidly and deeply anesthetized with chloral hydrate (250 mg/kg, *i.p.*) and perfused through the heart with phosphate buffered saline (PBS), followed by ice-cold 4% paraformaldehyde in 0.1 M phosphate buffer (PB). Brains were removed and postfixed in 4% paraformaldehyde-PB for 12 hours and then cryoprotected in 20% glycerol-0.1 M PB for 48–72 hours. Free-floating sections (40 *μ*m) containing the LA or MGm/PIN were cut using a sliding microtome. After blocked in PBS containing 1% BSA (Sigma Fraction V, Cat. No. A-3059), slices were incubated overnight at room temperature in either anti-Arc/Arg3.1 antibody (mouse monoclonal, 1 : 500; Santa Cruz Biotechnology), anti-EGR-1 antibody (mouse polyclonal, 1 : 1000; Santa Cruz Biotechnology), or anti-c-Fos antibody (rabbit polyclonal, 1 : 1000; Cell Signaling) in PBS with 1% BSA. After extensive washes in PBS, tissue sections were visualized using VectaStain ABC kit (Vector Laboratories) and developed in DAB peroxidase substrate (Sigma) for 5 min. Sections were mounted on Fisherbrand electrostatic slides and coverslipped. Sections from comparable anterior-posterior levels were selected for scoring (LA: *∼*3.2-3.3 mm posterior to Bregma; MGm/PIN: *∼*5.6-5.7 mm posterior to Bregma), Cell counts were taken from at least 3 sections for LA and MGm/PIN per rat and scored using a defined boundary roughly equivalent to the size of the LA or MGm/PIN using NIH ImageJ. Because every sixth section through the amygdala was processed for immunohistochemistry, it was not necessary to correct for double-counting. For analysis, cell counts were averaged into a single score for each rat. Counts from the ipsilateral side were expressed as a percentage of that on the contralateral side, and data were then analyzed using paired-sample *t*-test. 

## 3. Results

### 3.1. LTP-Inducing Stimulation of Thalamo-LA Synapses Regulates ERK Activation in Both the LA and the MGm/PIN

**Figure 1:**
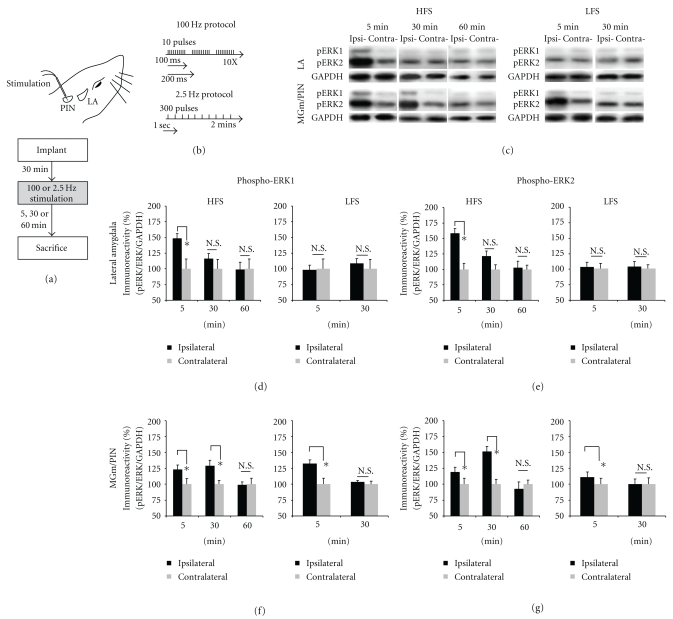
High-frequency stimulation of the MGm/PIN promotes ERK phosphorylation in LA at 5 min and in the MGm/PIN at 30 min after stimulation. (a) Placement of stimulation electrode and schematic representation of the experimental protocol. (b) Schematic representation of the HFS and LFS stimulation protocols. Anesthetized rats were given HFS or LFS and sacrificed at 5 min, 30 min or 60 min after stimulation. (c) Images of Western blots for phospho-ERK1/2 and associated GAPDH loading controls from LA (upper) and MGm/PIN (lower) samples after HFS or LFS. (d-e) Mean (±SEM) percent phospho-ERK1/2 immunoreactivity from LA punches taken from rats receiving HFS (left) or LFS (right) and sacrificed at 5 min (HFS: *n* = 6; LFS: *n* = 6), 30 min (HFS: *n* = 6; LFS: *n* = 8), or 60 min (*n* = 6). (f-g) Mean (±SEM) percent phospho-ERK1/2 immunoreactivity from MGm/PIN punches taken from rats receiving HFS (left) or LFS (right) and sacrificed at 5 min (HFS: *n* = 6; LFS: *n* = 6), 30 min (HFS: *n* = 5; LFS: *n* = 5), or 60 min (*n* = 6). For each figure, phospho-ERK1/2 levels have been normalized to total-ERK1/2 levels for each sample and counts on the ipsilateral (stimulated) side have been expressed as a percentage of those on the contralateral (nonstimulated) side. *P < .05 relative to the ipsilateral side N.S. = not significant.

Our lab has recently shown that HFS of the thalamo-LA pathway regulates ERK phosphorylation in the LA and that pharmacological blockade of ERK activation in the LA impairs LTP at thalamo-LA synapses, *in vivo* [22]. Interestingly, ERK activation at the level of the MGm/PIN also appears to be critical for LTP at thalamo-LA synapses; intra-MGm/PIN infusion of a MEK inhibitor also impairs LTP in the thalamo-LA pathway [23]. This pattern of findings collectively suggests that LTP at thalamo-LA synapses regulates ERK activation in both the LA and the MGm/PIN. In the present experiment, we tested this hypothesis by examining phospho-ERK in both LA and MGm/PIN in anesthetized rats after LTP-inducing stimulation of the thalamo-LA pathway, *in vivo *(Figure 1(a)). Rats were given 100 Hz HFS of the MGm/PIN (Figure 1(b)), a protocol that induces a reliable LTP at thalamo-LA synapses [22, 25, 26]. Control rats received 2.5 Hz LFS (Figure 1(b)), a protocol that does not induce LTP [22]. Rats were then sacrificed at different time points after stimulation (5 min, 30 min, or 60 min).

The findings for the LA are presented in Figures 1(d)-1(e), while images of Western blots are presented in Figure 1(c) (top). Consistent with our previous findings [22], rats receiving HFS of the thalamo-LA pathway exhibited significantly elevated levels of both phospho-ERK1 (Figure 1(d)) and phospho-ERK2 (Figure 1(e)) in the LA 5 min after stimulation (pERK1: *t*(5) = 3.871, *P* < .05; pERK2: *t*(5) = 3.631, *P* < .05; Figures 1(d)-1(e), left). No significant differences were observed for the 30 (pERK1: *t*(5) = 2.026, *P* > .05; pERK2: *t*(5) = 1.425, *P* > .05] or 60 min [pERK1: *t*(5) = 0.254, *P* > .05; pERK2: *t*(5) = 0.268, *P* > .05) time points (Figures 1(d)-1(e), left). Importantly, this effect was not accounted for by changes in the total amount of ERK1 or ERK2 protein (data not shown); no significant differences were observed for the 5 min (ERK1: *t*(5) = 1.268, *P* > .05; ERK2: *t*(5) = 0.882, *P* > .05), 30 min [ERK1: *t*(5) = 0.369, *P* > .05; ERK2: *t*(5) = 0.542, *P* > .05], or 60 min (ERK1: *t*(5) = 1.847, *P* > .05; ERK2: *t*(5) = 0.434, *P* > .05) time points. Further, this effect was not observed in LFS controls (Figures 1(d)-1(e), right); no significant differences were observed for either the 5 min (pERK1: *t*(5) = 0.725, *P* > .05; pERK2: *t*(5) = 0.759, *P* > .05), or 30 min (pERK1: *t*(7) = 1.076, *P* > .05; pERK2: *t*(7) = 0.300, *P* > .05) time points following LFS. 

The findings for the MGm/PIN are presented in Figures 1(f)-1(g), while images of Western blots are presented in Figure 1(c) (bottom). We found that ERK activation was enhanced in the MGm/PIN following HFS, but with a different time course from that in the LA. Rats receiving HFS of the thalamo-LA pathway exhibited significantly elevated levels of both phospho-ERK1 (Figure 1(f)) and phospho-ERK2 (Figure 1(g)) in the MGm/PIN at both 5 min (pERK1: *t*(5) = 2.897, *P* < .05; pERK2: *t*(5) = 2.596, *P* < .05) and 30 min after stimulation (pERK1: *t*(3) = 5.655, *P* < .05; pERK2: *t*(4) = 3.747, *P* < .05). No significant differences were observed for the 60 min time point (pERK1: *t*(4) = 0.405, *P* > .05; pERK2: *t*(4) = 0.073, *P* > .05) (Figures 1(f)-1(g), left). Importantly, the increase in phospho-ERK at both time points was not accounted for by changes in the total amount of ERK1 or ERK2 protein (not shown); no significant differences were observed for the 5 min (ERK1: *t*(5) = 0.520, *P* > .05; ERK2: *t*(5) = 0.731, *P* > .05), 30 min (ERK1: *t*(4) = 0.661, *P* > .05; ERK2: *t*(4) = 0.648, *P* > .05), or 60 min (ERK1: *t*(4) = 1.131, *P* > .05; ERK2: *t*(4) = 0.195, *P* > .05) time points. Interestingly, LFS controls were also observed to have elevated levels of phospho-ERK1 and phospho-ERK2 in MGm/PIN at 5 minutes (pERK1: *t*(5) = 3.193, *P* < .05; pERK2: *t*(5) = 4.073, *P* < .05), but not at 30 min (pERK1: *t*(5) = 0.529, *P* > .05; pERK2: *t*(5) = 0.067, *P* > .05) (Figures 1(f)-1(g), right). Further, no significant differences in total ERK were observed (data not shown) for the 5 min (ERK1: *t*(5) = 1.551, *P* > .05; ERK2: *t*(5) = 0.691, *P* > .05) or 30 min (ERK1: *t*(4) = 0.224, *P* > .05; ERK2: *t*(4) = 1.611, *P* > .05) time points following LFS. The fact that both HFS and LFS induced increases in ERK activation in the MGm/PIN at 5 min suggests that the elevated ERK phosphorylation in MGm/PIN at this time point was due to local electrical stimulation. Importantly, at the 30 min time point only HFS promoted significant ERK activation in the MGm/PIN, suggesting that the enhanced ERK phosphorylation in MGm/PIN at this later time point is specifically associated with LTP. 

### 3.2. LTP-Inducing Stimulation of Thalamo-LA Synapses Regulates ERK-Driven IEG Expression in Both the LA and the MGm/PIN

**Figure 2:**
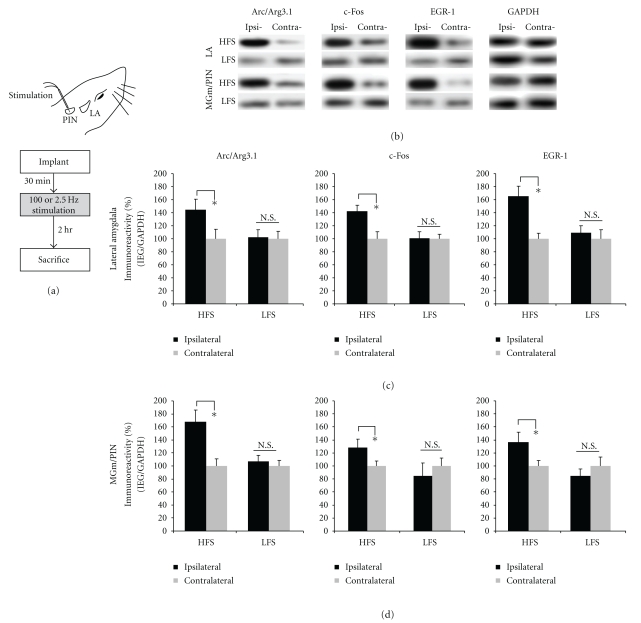
High-frequency stimulation of the MGm/PIN promotes ERK-driven IEG expression in both the LA and MGm/PIN. (a) Placement of stimulation electrode and schematic of the experimental protocol. Rats were given HFS or LFS and sacrificed 2 hours after stimulation. (b) Images of Western blots for Arc/Arg3.1, c-Fos, EGR-1, and GAPDH from both LA (top) and MGm/PIN samples (bottom). (c) Mean (±SEM) percent IEG immunoreactivity from LA punches taken from rats given HFS (*n* = 9) or LFS (*n* = 9). (d) Mean (±SEM) percent IEG immunoreactivity from MGm/PIN punches taken from rats given HFS (*n* = 9) or LFS (*n* = 9). In each figure, IEG levels have been normalized to GAPDH for each sample, and IEG expression on the ipsilateral side has been expressed as a percentage of that on the contralateral side for each rat. *P < .05 relative to the ipsilateral side N.S. = not significant.

**Figure 3:**
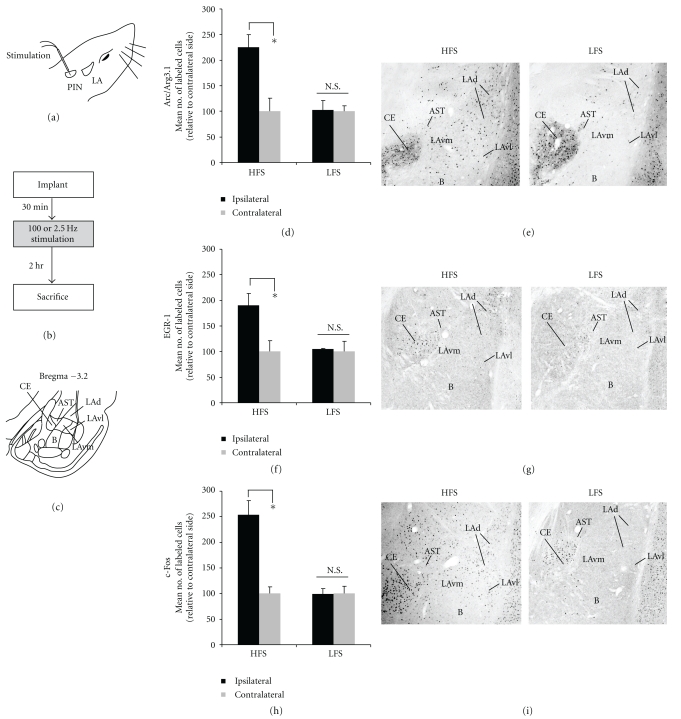
High-frequency stimulation of the MGm/PIN promotes increased immunolabeling of ERK-driven IEGs in the LA (a) Placement of stimulation electrode. (b) Schematic of experimental protocol. Rats were given HFS or LFS and sacrificed 2 hours after stimulation. (c) Schematic of the amygdala at Bregma −3.2. (d) Mean (±SEM) percent Arc/Arg3.1 immunoreactive cells in the LA from rats receiving HFS (*n* = 6) or LFS (*n* = 6). (e) Photomicrographs showing Arc/Arg3.1-labeled cells from rats receiving HFS (left) or LFS (right). (f) Mean (±SEM) percent EGR-1 immunoreactive cells in the LA from rats receiving HFS (*n* = 6) or LFS (*n* = 6). (g) Photomicrographs showing EGR-1-labeled cells from rats receiving HFS (left) or LFS (right). (h) Mean (±SEM) percent c-Fos immunoreactive cells in the LA from rats receiving HFS (*n* = 6) or LFS (*n* = 6). (i) Photomicrographs showing c-Fos-labeled cells from rats receiving HFS (left) or LFS (right). In each experiment, ipsilateral cell counts have been expressed as a percentage of contralateral cell counts for each rat. *P < .05 relative to the ipsilateral side N.S. = not significant. LAd = dorsal division of the lateral amygdala; LAv l = ventrolateral division of the lateral amygdala; LAvm = ventromedial division of the lateral amygdala; CE = central amygdala; B = basal amygdala; AST = amygdala-striatal transition zone.

**Figure 4:**
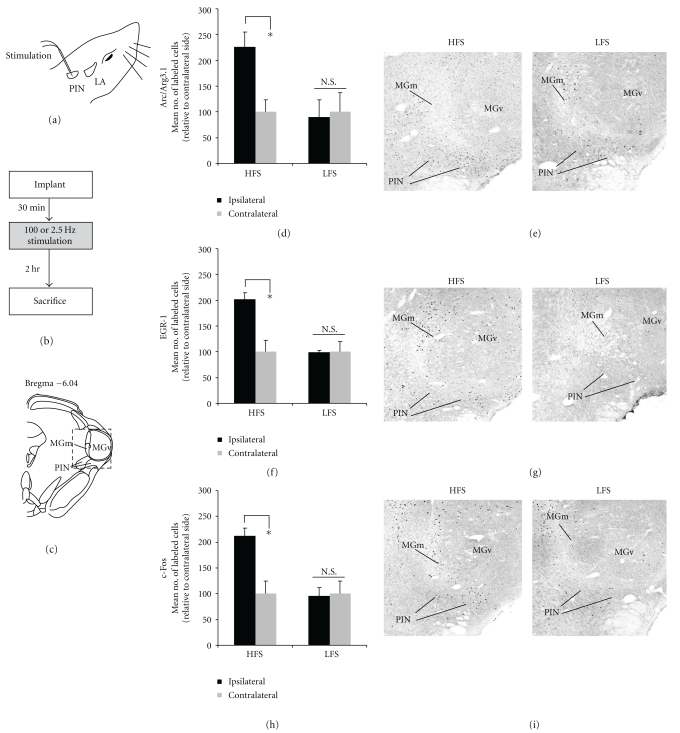
High-frequency stimulation of the MGm/PIN promotes increased immunolabeling of ERK-driven IEGs in the MGm/PIN. (a) Placement of stimulation electrode. (b) Schematic representation of experimental protocol. Rats were given HFS or LFS and sacrificed 2 hours after stimulation. (c) Schematic representation of the auditory thalamus at Bregma –5.6. (d) Mean (±SEM) percent Arc/Arg3.1 immunoreactive cells in the MGm/PIN from rats receiving HFS (*n* = 6) or LFS (*n* = 6). (e) Photomicrographs showing Arc/Arg3.1-labeled cells from rats receiving HFS (left) or LFS (right). (f) Mean (±SEM) percent EGR-1 immunoreactive cells in the MGm/PIN from rats receiving HFS (*n* = 6) or LFS (*n* = 6). (g) Photomicrographs showing EGR-1-labeled cells from rats receiving HFS (left) or LFS (right). (h) Mean (±SEM) percent c-Fos immunoreactive cells in the MGm/PIN from rats receiving HFS (*n* = 6) or LFS (*n* = 6). (i) Photomicrographs showing c-Fos-labeled cells from rats receiving HFS (left) or LFS (right). In each experiment, ipsilateral cell counts have been expressed as a percentage of contralateral cell counts for each rat. *P < .05 relative to the ipsilateral side N.S. = not significant. MGm = medial division of the medial geniculate nucleus; MGv = ventral division of the medial geniculate nucleus; PIN = posterior intralaminar nucleus.

In our first series of experiments, we observed significant increases in ERK activation in the LA and MGm/PIN following HFS of the thalamo-LA pathway. In the present experiments, we used a combination of Western blotting and immunohistochemistry to examine whether LTP-inducing stimulation of thalamic input synapses to the LA regulate the ERK-driven IEGs Arc/Arg3.1, EGR-1 and c-Fos in the LA and the MGm/PIN. As before, anesthetized rats received either HFS or LFS of thalamic inputs to the LA and were sacrificed by decapitation 2 hours later (Figures 2(a), 3(a), 4(a)), a time point which is sufficient for observing IEG expression in the LA after LTP [27]. 

The results of our Western blotting experiments are depicted in Figures 2(c)-2(d), while images of Western blots for LA and MGm/PIN are presented in Figure 2(b) (top and bottom, respectively). Western blotting revealed that HFS of the thalamo-LA pathway promoted significant elevations in the expression of Arc/Arg3.1, c-Fos, and EGR-1 protein expression in LA homogenates (Arc/Arg3.1: *t*(8) = 6.502, *P* < .05; c-Fos: *t*(8) = 3.901, *P* < .05; EGR-1: *t*(8) = 5.273, *P* < .05; Figure 2(c)). LFS, in contrast, had no significant effect on the expression of the three IEGs [Arc/Arg3.1: *t*(8) = 1.294, *P* > .05; c-Fos: *t*(8) = 0.241, *P* > .05; EGR-1: *t*(8) = 2.822, *P* > .05; Figure 2(c)]. A similar effect was also observed in the MGm/PIN (Figure 2(d)). As in the LA, HFS of the thalamo-LA pathway promoted significant elevations in the expression of Arc/Arg3.1, c-Fos, and EGR-1 protein expression in MGm/PIN homogenates [Arc/Arg3.1: *t*(8) = 3.642, *P* < .05; c-Fos: *t*(5) = 3.403, *P* < .05; EGR-1: *t*(6) = 2.59, *P* < .05; Figure 2(d)], while LFS had no effect [Arc/Arg3.1: *t*(8) = 1.163. *P* > .05; c-Fos: *t*(5) = 0.094, *P* > .05; EGR-1: *t*(6) = 1.720, *P* > .05; Figure 2(d)]. 

Immunohistochemical localization of the three IEGs after HFS and LFS in both the LA and MGm/PIN can be seen in Figures 3 and 4, respectively. Consistent with recent work in our laboratory [27], HFS induced robust expression of Arc/Arg3.1 in the LA [*t*(5) = 6.894, *P* < .05; Figure 3(d)], while LFS had no significant effect [*t*(5) = 0.124, *P* > .05; Figure 3(d)]. Arc/Arg3.1 cells were prominent in both the dorsal LAd and extending into the more ventral portions of the LAd and LAvl (Figure 3(e), left). In contrast, LFS produced little Arc/Arg3.1 expression in the LA (Figure 3(e), right). The analysis of EGR-1 and c-Fos revealed similar findings. For each protein, HFS induced significant expression in the LA (EGR-1: *t*(5) = 3.822, *P* < .05; c-Fos: *t*(5) = 4.144, *P* < .05; Figures 3(f)–3(h)), while LFS had no significant effect (EGR-1: *t*(5) = 0.709, *P* > .05; c-Fos: *t*(5) = 0.121, *P* > .05; Figures 3(f), 3(h)]. In contrast to Arc/Arg3.1, the distribution of EGR-1 labeled cells was much higher in the LAd relative to the LAv (Figure 3(g)), while c-Fos labeled cells were evenly scattered throughout the LA (Figure 3(i)).

In the MGm/PIN, HFS induced significant expression of Arc/Arg3.1 [*t*(6) = 22.556, *P* < .05], EGR-1 [*t*(5) = 13.470, *P* < .05], and c-Fos [*t*(5) = 10.959, *P* < .05] proteins (Figures 4(d), 4(f), 4(h)), while LFS did not [Arc/Arg3.1: *t*(5) = 1.913, *P* > .05; EGR-1: *t*(5) = 0.227, *P* > .05; c-Fos: *t*(5) = 0.395, *P* > .05]. Rats receiving HFS exhibited Arc/Arg3.1, EGR-1, and c-Fos labeled cells throughout the MGm and PIN, while very few labeled cells were observed in the MGv (Figures 4(e), 4(g), 4(i)).

### 3.3. NMDAR-Driven Synaptic Plasticity and NO Signaling in the LA Promote ERK Activation in Both the LA and the MGm/PIN Following LTP-Inducing Stimulation at Thalamo-LA Synapses

Our findings thus far indicate that LTP-inducing stimulation of thalamo-LA synapses is accompanied by ERK activation and ERK-driven gene expression in both the LA and the MGm/PIN. The HFS-induced activation of ERK-driven transcriptional regulation in MGm/PIN neurons is consistent with previous work that has shown that infusion of a MEK inhibitor into the MGm/PIN impairs LTP in the LA [23], a finding which suggests that LTP-induced activation of ERK in MGm/PIN neurons may contribute to presynaptic aspects of plasticity at the level of the LA. If so, might synaptic plasticity and NO signaling within the LA at the time of LTP induction be driving these changes at the level of the MGm/PIN? 

**Figure 5:**
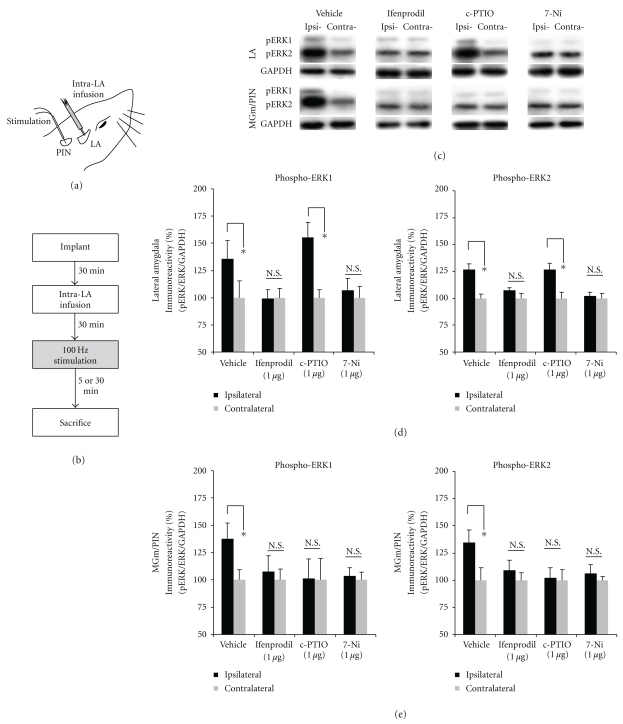
Pharmacological blockade of NMDAR-driven synaptic plasticity or NOS activation in the LA impairs ERK activation in both the LA and MGm/PIN following HFS, while blockade of extracellular NO impairs ERK activation in the MGm/PIN but not LA. (a) Placement of stimulation electrode and infusion cannula. (b) Schematic representation of the experimental protocol. Rats were given intra-LA infusion of the vehicle or drug (1 *μ*g/side) followed 30 min later by HFS of the MGm/PIN. Rats were sacrificed at 5 min (for the LA group) or 30 min (for the MGm/PIN group) following stimulation. (c) Images of Western blots for phospho-ERK1/2 and associated GAPDH controls from LA (top) and MGm/PIN (bottom) samples. (d) Mean (±SEM) percent phospho-ERK1/2 immunoreactivity from LA punches taken from rats given intra-LA infusions of 50% DMSO (vehicle; *n* = 5), 1 *μ*g/side ifenprodil (*n* = 5), 7-Ni (*n* = 6) or c-PTIO (*n* = 5). (e) Mean (±SEM) percent phospho-ERK1/2 immunoreactivity from MGm/PIN punches taken from rats given intra-LA infusions of 50% DMSO (vehicle; *n* = 5), 1 *μ*g/side ifenprodil (*n* = 6), 7-Ni (*n* = 6) or c-PTIO (*n* = 5). For each figure, phospho-ERK1/2 levels have been normalized to total-ERK1/2 levels for each sample and counts on the ipsilateral side have been expressed as a percentage of those on the contralateral side.*P < .05 relative to the ipsilateral side N.S. = not significant

In the present experiment, we asked whether blockade of NMDAR-driven synaptic plasticity and NO signaling in the LA impairs HFS-induced activation of ERK in both the LA and the MGm/PIN. Anesthetized rats were given intra-LA infusion of the NR2B-selective inhibitor ifenprodil, the NOS inhibitor 7-Ni, or the membrane impermeable scavenger of NO c-PTIO prior to LTP-inducing stimulation of the thalamo-LA pathway (Figure 5(a)). To examine the pharmacological regulation of ERK activation in the LA and the MGm/PIN, rats were sacrificed at either 5 or 30 min following stimulation, time points that we showed to be optimal for observing ERK activation in the LA and MGm/PIN following HFS, resp. (Figure 1). 

The findings are depicted in Figures 5(d)-5(e), while images of Western blots are presented in Figure 5(c) for LA and MGm/PIN (top and bottom, respectively). We observed a significant increase in phospho-ERK1/2 activation in the LA 5 min after HFS in vehicle-infused controls [pERK1: *t*(4) = 4.075, *P* < .05; pERK2: *t*(4) = 3.904, *P* < .05; Figure 5(d)]. Intra-LA infusion of either ifenprodil [pERK1: *t*(4) = 0.173, *P* > .05; pERK2: *t*(4) = 1.273, *P* > .05] or 7-Ni [pERK1: *t*(5) = 1.772, *P* > .05; pERK2: *t*(5) = 0.332, *P* > .05], however, significantly impaired HFS-induced ERK activation in the LA (Figure 5(d)). Interestingly, intra-LA infusion of c-PTIO had no effect on HFS-induced ERK activation in the LA [pERK1: *t*(4) = 5.026, *P* < .05; pERK2: *t*(4) = 3.640, *P* < .05; Figure 5(d)], indicating that blockade of extracellular NO within the LA has no effect on NO signaling within LA cells. 

In the MGm/PIN, we observed a significant increase in phospho-ERK1/2 activation 30 min after HFS in vehicle-infused controls [pERK1: *t*(4) = 3.035, *P* < .05; pERK2: *t*(4) = 3.864, *P* < .05; Figure 5(e)]. In contrast to the findings in the LA, however, infusion of all three drugs impaired HFS-induced ERK activation in the MGm/PIN (Figure 5(e)). Significant impairments in ERK activation were observed in the MGm/PIN following intra-LA infusion of either ifenprodil [pERK1: *t*(5) = 1.405, *P* > .05; pERK2: *t*(5) = 1.348, *P* > .05], 7-Ni [pERK1: *t*(5) = 0.812, *P* > .05; pERK2: *t*(5) = 0.302, *P* > .05], or c-PTIO [pERK1: *t*(4) = 0.487, *P* > .05; pERK2: *t*(4) = 0.193, *P* > .05]. 

Thus, blockade of NMDAR-driven synaptic plasticity and NO signaling at the level of the LA impairs HFS-induced ERK activation not only in the LA, but also in the MGm/PIN. Further, extracellular release of NO in the LA is required for HFS-induced ERK activation in the MGm/PIN, but not in the LA. 

### 3.4. NMDAR-Driven Synaptic Plasticity and NO-cGMP-PKG Signaling in the LA Promote ERK-Driven IEG Expression in Both the LA and the MGm/PIN after LTP-Inducing Stimulation of Thalamo-LA Synapses

**Figure 6:**
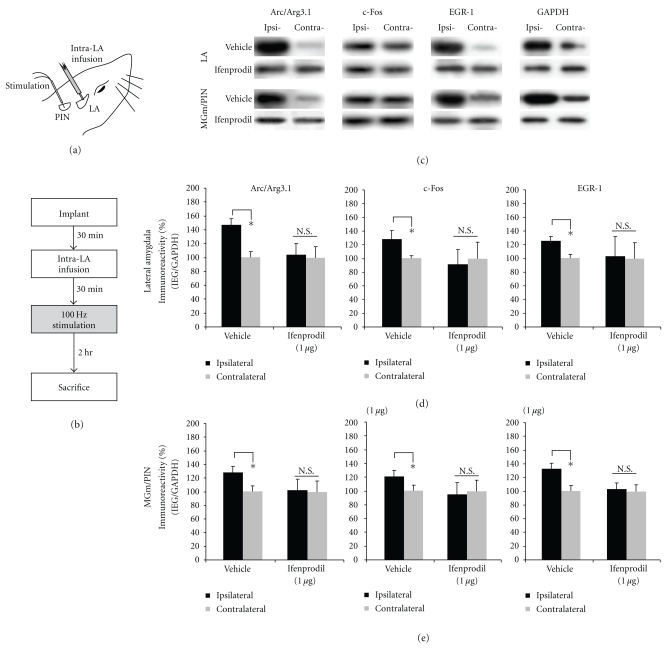
Pharmacological blockade of NMDAR-driven synaptic plasticity impairs ERK-driven IEG expression in both the LA and MGm/PIN following HFS. (a) Placement of stimulation electrode and infusion cannula. (b) Schematic representation of experimental protocol. Rats were given intra-LA infusion of the vehicle or 1 *μ*g ifenprodil followed 30 min later by HFS of the MGm/PIN. Rats were sacrificed 2 hours after stimulation. (c) Images of Western blots for Arc/Arg3.1, c-Fos, EGR-1 and GAPDH from both LA (top) and MGm/PIN (bottom) samples. (d) Mean (±SEM) percent Arc/Arg3.1, c-Fos and EGR-1 immunoreactivity from LA punches taken from rats given intra-LA infusion of 2% HBC-saline (vehicle; *n* = 8) or 1 *μ*g/side ifenprodil (*n* = 8). (e) Mean (±SEM) percent Arc/Arg3.1, c-Fos and EGR-1 immunoreactivity from MGm/PIN punches taken from rats given intra-LA infusion of 2% HBC-saline (vehicle; *n* = 8) or 1 *μ*g/side ifenprodil (*n* = 8). In each figure, IEG levels have been normalized to GAPDH for each sample, and IEG expression on the ipsilateral side has been expressed as a percentage of that on the contralateral side for each rat. *P < .05 relative to the ipsilateral side N.S. = not significant.

In this series of experiments, we examined whether blockade of NMDAR-driven synaptic plasticity and NO signaling in the LA impairs HFS-induced expression of the IEGs Arc/Arg3.1, c-Fos, and EGR-1 in both the LA and the MGm/PIN. As in the previous experiment, anesthetized rats were given intra-LA infusion of ifenprodil, 7-Ni, or c-PTIO prior to LTP-inducing stimulation of the thalamo-LA pathway (Figure 6(a)). In addition, we also ran a group that was infused with the protein kinase G (PKG) inhibitor Rp-8-Br-PET-cGMPS. To examine the pharmacological regulation of IEG expression in the LA and the MGm/PIN, rats were sacrificed 2 hrs following stimulation, a time point that we showed to be sufficient for observing HFS-induced IEG expression in the LA and MGm/PIN. 

The findings for rats infused with ifenprodil are depicted in Figures 6(d)-6(e), while images of Western blots are presented in Figure 6(c). We observed significant elevations in the expression of Arc/Arg3.1, c-Fos, and EGR-1 protein expression in LA homogenates from vehicle-infused controls [Arc/Arg3.1: *t*(7) = 3.440. *P* < .05; c-Fos: *t*(7) = 2.405, *P* < .05; EGR-1: *t*(7) = 5.009, *P* < .05; Figure 6(d)]. In contrast, those rats given intra-LA infusion of ifenprodil exhibited significantly impaired IEG expression in the LA [Arc/Arg3.1: *t*(7) = 0.377. *P* > .05; c-Fos: *t*(7) = 0.708, *P* > .05; EGR-1: *t*(7) = 0.306, *P* > .05; Figure 6(d)]. A similar pattern of findings was observed in the MGm/PIN (Figure 6(e)). Vehicle-infused controls exhibited significant elevations in the expression of Arc/Arg3.1, c-Fos, and EGR-1 protein expression in MGm/PIN homogenates [Arc/Arg3.1: *t*(7) = 2.629. *P* < .05; c-Fos: *t*(7) = 3.783, *P* < .05; EGR-1: *t*(7) = 3.440, *P* < .05; Figure 6(e)], while ifenprodil-infused rats exhibited significantly impaired IEG expression in the MGm/PIN [Arc/Arg3.1: *t*(7) = 0.211. *P* > .05; c-Fos: *t*(7) = 0.592, *P* > .05; EGR-1: *t*(7) = 0.139, *P* > .05; Figure 6(e)].

**Figure 7:**
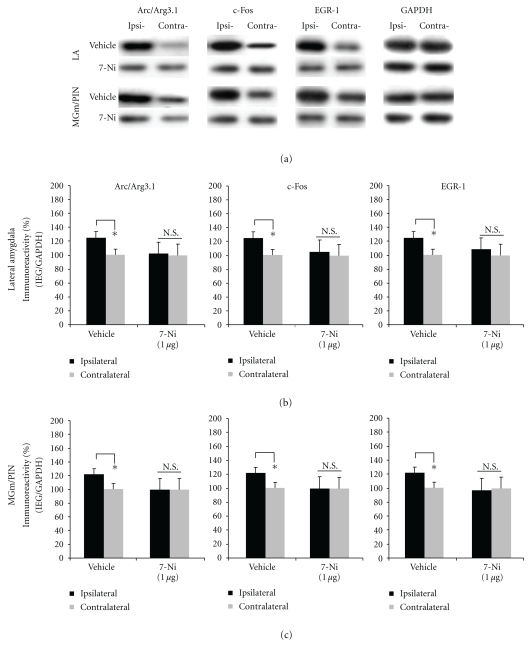
Pharmacological blockade of NOS activation in the LA impairs ERK-driven IEG expression in both LA and MGm/PIN following HFS. (a) Images of Western blots for Arc/Arg3.1, c-Fos, EGR-1 and GAPDH from both LA (top) and MGm/PIN (bottom) samples. (b) Mean (±SEM) percent Arc/Arg3.1, c-Fos and EGR-1 immunoreactivity from LA punches taken from rats given intra-LA infusion of 50% DMSO (vehicle; *n* = 8) or 1 *μ*g/side 7-Ni (*n* = 8). (c) Mean (±SEM) percent Arc/Arg3.1, c-Fos and EGR-1 immunoreactivity from MGm/PIN punches taken from rats given intra-LA infusion of 50% DMSO (vehicle; *n* = 8) or 1 *μ*g/side 7-Ni (*n* = 8). In each figure, IEG levels have been normalized to GAPDH for each sample, and IEG expression on the ipsilateral side has been expressed as a percentage of that on the contralateral side for each rat. *P < .05 relative to the ipsilateral side N.S. = not significant.

The findings for rats infused with 7-Ni are depicted in Figures 7(b)-7(c), and images of Western blots are presented in Figure 7(a). We observed significant elevations in the expression of Arc/Arg3.1, c-Fos, and EGR-1 protein expression in LA homogenates in vehicle-infused controls [Arc/Arg3.1: *t*(7) = 2.374. *P* < .05; c-Fos: *t*(7) = 2.462, *P* < .05; EGR-1: *t*(7) = 2.402, *P* < .05; Figure 7(b)]. In contrast, those rats given intra-LA infusion of 7-Ni exhibited significantly impaired IEG expression in the LA [Arc/Arg3.1: *t*(7) = 0.672. *P* > .05; c-Fos: *t*(7) = 1.101, *P* > .05; EGR-1: *t*(7) = 1.499, *P* > .05; Figure 7(b)]. A similar pattern of findings was observed in the MGm/PIN (Figure 7(c)). Vehicle-infused controls exhibited significant elevations in the expression of Arc/Arg3.1, c-Fos, and EGR-1 protein expression in MGm/PIN homogenates [Arc/Arg3.1: *t*(7) = 3.066. *P* < .05; c-Fos: *t*(7) = 2.383, *P* < .05; EGR-1: *t*(7) = 2.687, *P* < .05; Figure 7(c)], while 7-Ni-infused rats exhibited significantly impaired IEG expression in the MGm/PIN [Arc/Arg3.1: *t*(7) = 0.065. *P* > .05; c-Fos: *t*(7) = 0.025, *P* > .05; EGR-1: *t*(7) = 0.460, *P* > .05; Figure 7(c)].

**Figure 8:**
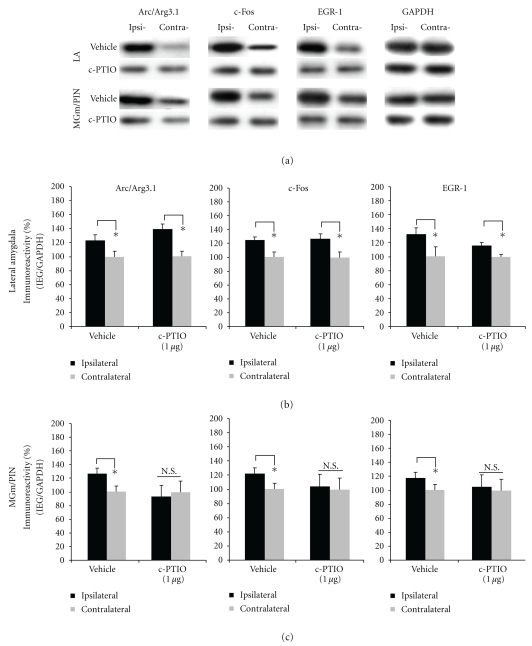
Pharmacological blockade of extracellular NO in the LA impairs ERK-driven IEG expression in the MGm/PIN, but not the LA, following HFS. (a) Images of Western blots for Arc/Arg3.1, c-Fos, EGR-1, and GAPDH from both LA (top) and MGm/PIN (bottom) samples. (b) Mean (±SEM) percent Arc/Arg3.1, c-Fos, and EGR-1 immunoreactivity from LA punches taken from rats given intra-LA infusion of 50% DMSO (vehicle; *n* = 8) or 1 *μ*g/side c-PTIO (*n* = 8). (C) Mean (±SEM) percent Arc/Arg3.1, c-Fos and EGR-1 immunoreactivity from MGm/PIN punches taken from rats given intra-LA infusion of 50% DMSO (vehicle; *n* = 8) or 1 *μ*g/side c-PTIO (*n* = 8). In each figure, IEG levels have been normalized to GAPDH for each sample, and IEG expression on the ipsilateral side has been expressed as a percentage of that on the contralateral side for each rat. *P < .05 relative to the ipsilateral side N.S. = not significant.

The findings for rats infused with c-PTIO are depicted in Figures 8(b)-8(c), with images of Western blots presented in Figure 8(a). We observed significant elevations in the expression of Arc/Arg3.1, c-Fos, and EGR-1 protein expression in LA homogenates from vehicle-infused controls [Arc/Arg3.1: *t*(7) = 2.374. *P* < .05; c-Fos: *t*(7) = 2.462, *P* < .05; EGR-1: *t*(7) = 2.402, *P* < .05; Figure 8(b)]. Consistent with our ERK data, those rats given intra-LA infusion of c-PTIO also exhibited elevated IEG expression in the LA [Arc/Arg3.1: *t*(7) = 2.545. *P* < .05; c-Fos: *t*(7) = 2.406, *P* < .05; EGR-1: *t*(7) = 2.509, *P* < .05; Figure 8(b)]. A different pattern of findings, however, was observed in the MGm/PIN (Figure 8(c)). Vehicle-infused controls exhibited significant elevations in the expression of Arc/Arg3.1, c-Fos, and EGR-1 protein expression in MGm/PIN homogenates [Arc/Arg3.1: *t*(7) = 3.066. *P* < .05; c-Fos: *t*(7) = 2.383, *P* < .05; EGR-1: *t*(7) = 2.687, *P* < .05; Figure 8(c)], while cPTIO-infused rats exhibited significantly impaired IEG expression in the MGm/PIN [Arc/Arg3.1: *t*(7) = 1.241. *P* > .05; c-Fos: *t*(7) = 0.696, *P* > .05; EGR-1: *t*(7) = 1.600, *P* > .05; Figure 8(c)].

**Figure 9:**
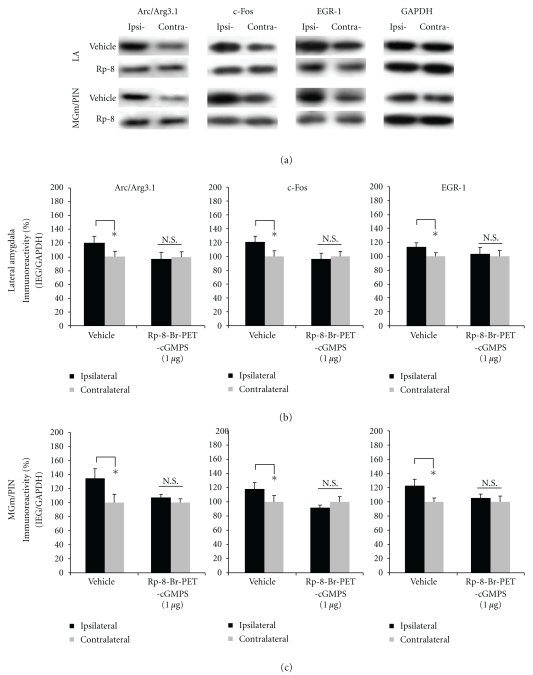
Pharmacological blockade of PKG in the LA impairs ERK-driven IEG expression in both LA and MGm/PIN following HFS. (a) Images of Western blots for Arc/Arg3.1, c-Fos, EGR-1, and GAPDH from both LA (top) and MGm/PIN (bottom) samples. (b) Mean (±SEM) percent Arc/Arg3.1, c-Fos, and EGR-1 immunoreactivity from LA punches taken from rats given intra-LA infusion of ACSF (vehicle; *n* = 8) or 1 *μ*g/side Rp-8-Br-PET-cGMPS (*n* = 8). (c) Mean (±SEM) percent Arc/Arg3.1, c-Fos and EGR-1 immunoreactivity from MGm/PIN punches taken from rats given intra-LA infusion of ACSF (vehicle; *n* = 8) or 1 *μ*g/side Rp-8-Br-PET-cGMPS (*n* = 8). In each figure, IEG levels have been normalized to GAPDH for each sample, and IEG expression on the ipsilateral side has been expressed as a percentage of that on the contralateral side for each rat. *P < .05 relative to the ipsilateral side N.S. = not significant.

The findings for rats infused with PKG inhibitor Rp-8-Br-PET-cGMPS are depicted in Figure 9(b)-9(c), and images of Western blots are presented in Figure 9(a). We observed significant elevations in the expression of Arc/Arg3.1, c-Fos, and EGR-1 protein expression in LA homogenates from vehicle-infused controls [Arc/Arg3.1: *t*(7) = 7.972. *P* < .05; c-Fos: *t*(7) = 3.686, *P* < .05; EGR-1: *t*(7) = 4.599, *P* < .05; Figure 9(b)]. In contrast, those rats given intra-LA infusion of Rp-8-Br-PET-cGMPS exhibited significantly impaired IEG expression in the LA [Arc/Arg3.1: *t*(6) = 1.688. *P* > .05; c-Fos: *t*(7) = 0.631, *P* > .05; EGR-1: *t*(7) = 1.287, *P* > .05; Figure 9(b)]. A similar pattern of findings was observed in the MGm/PIN (Figure 9(c)). Vehicle-infused controls exhibited significant elevations in the expression of Arc/Arg3.1, c-Fos, and EGR-1 protein expression in MGm/PIN homogenates [Arc/Arg3.1: *t*(7) = 7.972. *P* < .05; c-Fos: *t*(7) = 4.064, *P* < .05; EGR-1: *t*(7) = 4.901, *P* < .05; Figure 9(c)], while rats infused with the PKG inhibitor exhibited significantly impaired IEG expression in the MGm/PIN [Arc/Arg3.1: *t*(6) = 1.688. *P* > .05; c-Fos: *t*(7) = 1.101, *P* > .05; EGR-1: *t*(7) = 0.401, *P* > .05; Figure 9(c)].

Thus, similar to the findings of our ERK experiments, blockade of NMDAR-driven synaptic plasticity and NO signaling at the level of the LA impairs HFS-induced IEG expression not only in the LA but also in the MGm/PIN. Further, extracellular release of NO in the LA appears to be required for HFS-induced IEG expression in the MGm/PIN, but not in the LA

## 4. Discussion

Long-term potentiation (LTP) at thalamo-LA synapses has been proposed as a candidate cellular mechanism of the formation of auditory fear memories, yet little is known about the molecular mechanisms underlying LTP at this synapse. In the present study, we have examined the regulation of ERK and that of three different ERK-driven IEGs at both sides of the thalamo-LA synapse after LTP-inducing stimulation. We found that LTP-inducing stimulation at thalamo-LA synapses is accompanied by ERK activation and ERK-driven gene expression not only in the LA, but also in regions of the MGm/PIN that are presynaptic to the LA. Further, pharmacological disruption of either NMDAR-driven synaptic plasticity or NO-cGMP-PKG signaling at the level of the LA impairs ERK activation and IEG expression in each region. Collectively, these findings suggest that NMDAR-driven synaptic plasticity and NO signaling within the LA coordinately regulate ERK activation and ERK-driven gene expression in both the LA and the MGm/PIN following LTP induction at thalamo-LA synapses.

### 4.1. LTP-Inducing Stimulation of Thalamo-LA Inputs Regulates ERK Activation and ERK-Driven IEG Expression in the LA and MGm/PIN

A recent study in our lab showed that LTP-inducing stimulation of thalamo-LA inputs induces ERK activation in the LA, and that intra-LA infusion of ERK/MAPK inhibitor impairs LTP at thalamo-LA synapses [22]. However, ERK activation at the level of the MGm/PIN also appears to be critical for LTP at thalamo-LA synapses; intra-MGm/PIN infusion of a MEK inhibitor also impairs LTP in the thalamo-LA pathway [23]. In the present study, we, therefore, examined ERK activation in both the LA and MGm/PIN following LTP-inducing stimulation of thalamo-LA synapses. Consistent with our previous findings [22], we showed that phospho-ERK expression was enhanced in the LA 5 min after HFS compared to LFS controls; significant elevations in phospho-ERK were not evident at later time points. In the MGm/PIN, however, HFS-induced phospho-ERK was observed at both 5 and 30 min following stimulation. Importantly, only the 30 min time point differed significantly from LFS controls, suggesting that the increase observed within 5 min after HFS may have been due to nonspecific stimulation of the region alone. Thus, LTP-inducing stimulation of thalamo-LA synapses induces ERK activation in both the LA and MGm/PIN, but the activation in the MGm/PIN is delayed relative to that in the LA. 

Our findings also revealed that LTP-inducing stimulation of thalamo-LA synapses regulates the expression of the ERK-driven IEGs Arc/Arg3.1, c-Fos, and EGR-1 in both the LA and MGm/PIN. Previous studies have extensively documented the role of Arc/Arg3.1 [28–32] and EGR-1 [33–37] in hippocampal LTP, but little is known about the role of these IEGs in amygdala LTP. The Arc/Arg3.1 gene encodes for a synaptic activity-induced effecter protein and has been shown in previous studies to be required for LTP and hippocampal-dependent learning and memory [28]. Arc/Arg3.1 is known to be induced by patterns of neural activity that promote synaptic plasticity and is thought to be trafficked and localized to recently potentiated synapses [29–32]. In our own lab, we have recently shown that LTP induction at thalamo-LA synapses induces Arc/Arg3.1 mRNA and protein expression in the LA, and that antisense knockdown of Arc/Arg3.1 protein in the LA impairs memory consolidation of auditory fear conditioning; that is, LTM is impaired whereas STM is intact [27]. 

In contrast to Arc/Arg3.1, both EGR-1 and c-Fos are thought to behave as transcription factors, regulating the expression of late-response genes that are critical for long-term synaptic plasticity. Importantly, several studies have observed associative increases in the expression of c-Fos and EGR-1 in the LA after cued fear conditioning [38–40]. Furthermore, Malkani and colleagues showed that antisense knockdown of EGR-1 in the LA impairs memory formation of contextual fear conditioning [41], a finding that has recently been extended to auditory fear conditioning in our own lab [42]. Consistent with these findings, we report here that Arc/Arg3.1, c-Fos, and EGR-1 protein expression in the LA is enhanced after LTP-inducing stimulation of thalamic inputs to the LA, as well as in regions of the auditory thalamus that are presynaptic to the LA. Together with our ERK findings, these results suggest that ERK-driven transcriptional regulation at both sides of the thalamo-LA synapse may be critical for synaptic plasticity underlying the formation and/or consolidation of auditory fear memories. 

### 4.2. NMDAR-Driven Synaptic Plasticity and NO-cGMP-PKG Signaling Coordinately Regulate ERK Activation and ERK-Driven Gene Expression at Both Sides of the Thalamo-LA Synapse

Our findings of enhanced activation of ERK-driven transcriptional regulation in both LA and MGm/PIN neurons is consistent with previous work that has shown that infusion of a MEK inhibitor into either the LA or the MGm/PIN impairs LTP in the LA [22, 23] and suggests that LTP-induced activation of ERK in MGm/PIN and LA neurons may contribute to pre- and postsynaptic aspects of plasticity at the level of the LA, respectively. Long-term synaptic plasticity has long been thought to involve NMDAR-driven recruitment of intracellular signaling pathways that promote long-term plastic change and memory through alterations of transcription and translation and accompanying morphological changes at both pre- and postsynaptic sites [43–48]. Further, many studies have suggested that the NO-cGMP-PKG signaling pathway plays a critical role in coordinating these two events [49–54], behaving both as a regulator of transcription in the postsynaptic cell [50] as well as a “retrograde signal” that can promote enhanced release of transmitter in the presynaptic cell [55] as well as structural changes in the presynaptic terminal [56, 57]. 

While most widely studied in the hippocampus [49, 58–63] and cerebellum [58], recent evidence from our laboratory has suggested that NO signaling in the LA is also critical to fear memory formation [17]. Neuronal NOS (nNOS) is expressed in LA neurons and in postsynaptic sites of excitatory synapses in the LA [17]. Further, pharmacological manipulation of NO signaling in the LA using either a NOS inhibitor, a membrane-impermeable scavenger of NO, or a PKG inhibitor impairs memory consolidation of auditory fear conditioning and LTP at auditory thalamic input synapses to the LA, *in vitro* [17, 18].

In the present study, we used pharmacological methods to ask whether NMDAR-driven synaptic plasticity and NO-cGMP-PKG signaling may be regulating both ERK and ERK-driven transcription within the LA and the MGm/PIN. We showed that blockade of NR2B (via ifenprodil), NOS (via 7-Ni), or PKG (via Rp-8-Br-PET-cGMPS) significantly impaired HFS-induced activation of ERK and ERK-driven gene expression in both the LA and the MGm/PIN. Remarkably, however, blockade of extracellular NO signaling (via c-PTIO) significantly impaired HFS-induced activation of ERK and ERK-driven gene expression in the MGm/PIN, but not in the LA. These findings suggest that the ERK activation and downstream IEG expression in MGm/PIN following LTP is driven by NO “retrograde signaling” at the level of the LA. The identity of the signal that links NO release at the level of the LA with ERK activation and ERK-driven gene expression at the level of the MGm/PIN is currently unknown, as is how such a signal may propagate in a retrograde manner from synapse to nucleus across a *∼*2-3 mm distance in such a rapid manner. However, previous reports have suggested that retrograde transport can occur very rapidly in neurons, between *∼*4–8 mm/hr [64]. Further, while the mechanism by which NO-cGMP-PKG signaling within the LA promotes ERK activation and ERK-driven gene expression in the LA and MGm/PIN during LTP induction is presently unknown, previous studies have suggested that PKG or its downstream substrates can activate Raf-1, an upstream regulator of ERK1/2 [65], or inhibit protein phosphatase-1 [66], which may indirectly regulate ERK1/2. Further, NO-cGMP-PKG signaling has been shown to regulate CREB phosphorylation in the hippocampus following LTP induction, presumably by affecting upstream kinases such as ERK [50]. 

### 4.3. A Model of Synaptic Plasticity at Thalamo-LA Synapses

**Figure 10:**
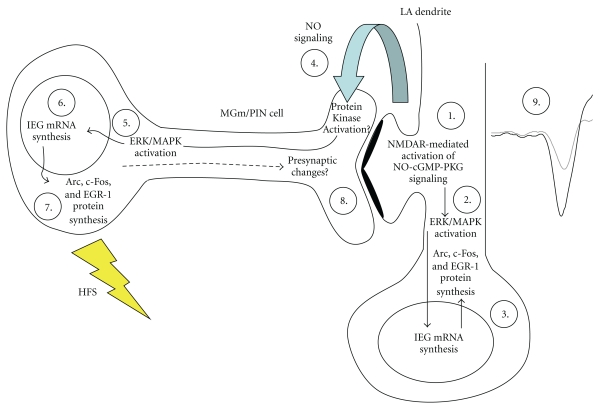
A model of the biochemical mechanisms underlying LTP at thalamo-LA synapses. See text for details

The present findings are consistent with a revised model of the molecular events underlying synaptic plasticity at thalamo-LA synapses in which NMDAR-driven synaptic plasticity and NO signaling in LA neurons promotes pre- and postsynaptic alterations at thalamo-LA synapses via regulation of ERK-driven gene expression in MGm/PIN and LA neurons, respectively, (Figure 10). In that model, thalamo-LA LTP is hypothesized to involve both pre- and postsynaptic modifications at thalamo-LA synapses. These modifications are first triggered by NMDAR-mediated activation of the NO-cGMP-PKG signaling pathway in the postsynaptic cell (Step 1) that promote the activation of ERK (Step 2)[22] and ERK-driven IEG expression (Step 3) in LA neurons. The transcription of these ERK-driven genes is ultimately thought to lead to postsynaptic functional and/or structural changes that contribute to long-term synaptic plasticity at this synapse [3]. Concurrently, “retrograde signaling” via NO (Step 4) may promote the activation of ERK (Step 5) and ERK-driven transcription (Step 6-7) in presynaptic thalamic targets of LA neurons that are necessary to promote structural and/or functional changes on the presynaptic side of LA synapses (Step 8). Together with the postsynaptic modifications driven by ERK signaling in the LA, these presynaptic modifications act to strengthen the connectivity of thalamo-LA synapses, which is reflected neurophysiologically in an enhanced response to the CS in the LA after LTP induction (Step 9). 

In support of this model, recent studies from our lab and others have shown that ERK-driven transcription in the MGm/PIN is required not only for fear memory consolidation [23, 67, 68] but also for synaptic plasticity at thalamo-LA synapses [23]. Further, auditory fear conditioning has recently been shown to lead to increased expression of the presynaptically localized proteins synaptophysin and synapsin in the LA [20, 21, 69], suggesting that fear memory consolidation is accompanied by presynaptic alterations at LA synapses. Finally, recent studies from our lab have shown that auditory fear conditioning is associated with increases in the activation of ERK and ERK-driven IEG expression in the LA and MGm/PIN [19, 21], and that knockdown of EGR-1 in MGm/PIN neurons impairs both fear memory consolidation and the training-induced expression of both synapsin and synaptophysin in the LA [21]. Together with the findings of the present paper, these findings collectively suggest that both synaptic plasticity at thalamo-LA synapses and memory consolidation of auditory fear conditioning are subserved by pre- and postsynaptic modifications at LA synapses.

## 5. Conclusions

In summary, our findings suggest that NMDAR-driven synaptic plasticity and NO signaling within the LA coordinately regulate ERK activation and ERK-driven gene expression in both the LA and the MGm/PIN following LTP induction at thalamo-LA synapses, and further suggest that synaptic plasticity in the LA promotes ERK-driven transcription in MGm/PIN neurons via NO-driven “retrograde signaling”. These findings further extend what is known about the molecular basis of LTP within the LA, and provide additional evidence that studying LTP at thalamo-LA synapses may inform us about the molecular basis of fear memory formation in the amygdala.
